# FIFA World Cup Qatar 2022^TM^ stadium patient evacuation: A system testing simulation-based exercise

**DOI:** 10.5339/qmj.2023.1

**Published:** 2022-12-30

**Authors:** Padarath Gangaram, Wayne Thomson, Brendon Morris, Guillaume Alinier

**Affiliations:** ^1^Hamad Medical Corporation Ambulance Service, Doha, Qatar. Address: HMC Ambulance Service Head Quarter, Al Ryyan Road, Medical City, P.O. Box 3050, Doha, Qatar. E-mail: pgangaram@hamad.qa; ^2^Durban University of Technology, Durban, South Africa.; ^3^University of Hertfordshire, Hatfield, UK.; ^4^Weill Cornell Medicine - Qatar, Doha, Qatar.; ^5^Northumbria University, Newcastle upon Tyne, UK.

**Keywords:** Ground ambulance transport, helicopter transport, mass gathering events, stadium evacuation, patient transport, HEMS, FIFA World Cup Qatar 2022^TM^.

## Abstract

Background: As the State of Qatar is soon to host the Federation International of Football Associations (FIFA) 2022 World Cup tournament, the health sector has also been preparing for the event to increase its capacity to meet the expected additional health demand. The readiness of the health sector is being tested and improved through a number of simulation-based exercises. In this case, it relates to testing in a realistic manner the complete evacuation process of a patient using two very different modes of transportation, from a distant FIFA stadium up to the handover phase in the main trauma center in the State of Qatar.

Method: In this controlled simulation-based pilot study, the total evacuation time of a patient from the 60,000-fan capacity Al Bayt Stadium (ABS), situated in a rural northern part of Qatar, to Hamad General Hospital (HGH) Trauma Resuscitation Unit (TRU) situated approximately 50 km away, was compared when transported by helicopter and by ambulance. The Scenario for the simulation was based on a player who sustained a fractured lower leg and a concussion during a football match and needed urgent evacuation from the ABS Players’ Medical Clinic near Al Khor to HGH in Doha. The same Scenario was enacted twice, the first time with a ground Hamad Medical Corporation Ambulance Service (HMCAS) ambulance and the second time with an HMCAS LifeFlight helicopter.

Results: The transportation phase for Scenario 2 (LifeFlight helicopter) was 63% faster than for Scenario 1 (ground ambulance). However, upon arrival at HGH, the patient arrived sooner at the TRU in Scenario 1 compared with Scenario 2. The overall mission time was thus only 6 minutes and 22 seconds faster by LifeFlight helicopter as compared with the ground ambulance.

Conclusions: According to this simulation-based pilot study, using a helicopter to transport patients 50 km from the ABS 2022 FIFA World Cup stadium to the HGH TRU was only marginally faster by helicopter than using a ground-based ambulance. In addition, the ambulance was not using emergency driving operations, which when used would have further reduced the time taken for the ambulance to reach HGH TRU. Therefore, having a helicopter on standby there would not significantly improve the transport time of a critically ill/injured patient’s access to definitive care and will not be available during the FIFA World Cup Qatar 2022^TM^ unless it is called upon to respond to a mass casualty incident or bring additional Critical Care Paramedic resources to the stadium.

## Introduction

International sporting competitions often attract thousands of spectators, especially international football tournaments.^1^ Large sporting events constitute mass gatherings that are associated with a number of health challenges as highlighted in several publications.^1,2^ Although significant resources are deployed to respond to the medical needs of athletes and spectators in terms of manpower and temporary health facilities, a proportion of patients may require urgent transportation to hospital or other specialized healthcare facility for definitive care.^3^ In the State of Qatar, patient transportation options are usually by road, using ambulances, or by air, using helicopter emergency medical services.

Responding to an emergency call in a football stadium during a match presents specific challenges because of the configuration of the space, the safety measures in place, and the crowd present at such events, which constitutes a mass gathering.^4,5^ The identified common modes of transportation both have advantages and limitations based on various factors.

As the State of Qatar is soon to host the Federation International of Football Associations (FIFA) World Cup Qatar 2022^TM^ tournament, the health sector has been preparing for the event to improve its capacity to fulfill the expected health demand. Qatar is a small country on the east coast of the Arabic peninsula with a population of over 2.6 million inhabitants.^6^ The presence of fans and visitors at the tournament is expected to cause a surge in the overall country’s population by around 1.7 million people, which will result in a significantly higher number of emergency calls and hospital visits.^7^

## Background

**Figure 1.**
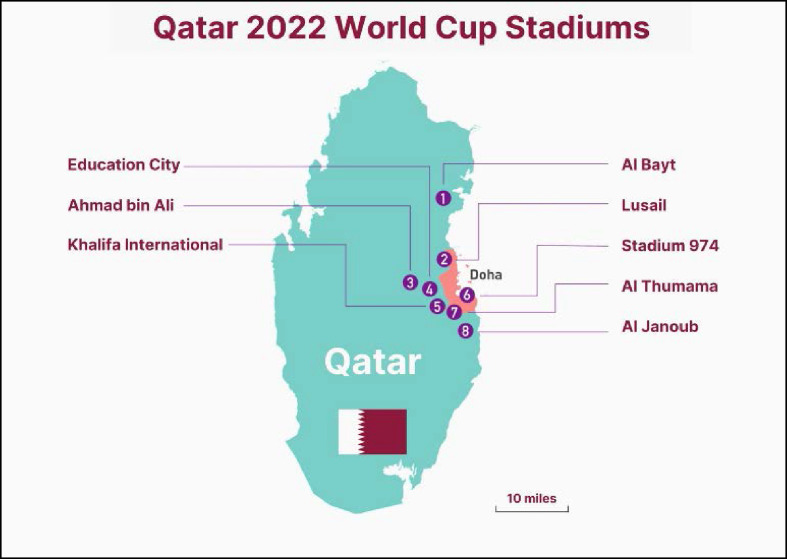
FIFA World Cup Qatar 2022^TM^ World Cup Stadia (Source: www.footyheadlines.com).

The present simulation-based controlled pilot study is based around the 60,000-fan capacity Al Bayt Stadium (ABS), situated in a rural northern part of Qatar, near Al Khor, approximately 50 km (Figure 1) away from Hamad General Hospital (HGH), which hosts the national Level 1 Trauma Center.^8^ The architectural design of ABS takes its inspiration from the traditional tents of the nomadic people of Qatar and the region alike. ABS is one of eight stadia for the FIFA World Cup Qatar 2022^TM^. It is the second largest stadium after Lusail Stadium, and the furthest from the Level 1 Trauma Center.^7^ ABS will be hosting nine games in total FIFA World Cup Qatar 2022^TM^, including six games during the group matches, one round of 16 games, a quarterfinal, and a semifinal, during the FIFA World Cup Qatar 2022^TM^. In addition, the stadium will also be hosting the opening ceremony and the Qatar national team’s opening game with Ecuador.

The planning and implementation of medical resources for the FIFA World Cup Qatar 2022^TM^ were tested during the FIFA Arab Cup (FAC) (November 17, 2021–December 18, 2021), which took place during the COVID-19 pandemic.^9^ Post-FAC, a review of the resources, staffing, venues, and vehicles were undertaken, and the medical resource allocation was finalized for the FIFA World Cup Qatar 2022^TM^, although this is always liable to change based on updated information as the event period approaches.

Medical resources allocated to ABS during operations will vary on match days and nonmatch days. A general clinic, staffed by a doctor and a nurse, will be operational between 6:00 am and 11:00 pm on nonmatch days in the inner security perimeter. In addition, a Qatar ambulance with two paramedics will be stationed outside the general clinic, in the inner security perimeter, 24 hours a day.

Based on the risk analysis profile, the medical operations at the stadium will be increased on match days to meet the expected demand, including but not limited to the following: stadium fire, road traffic accidents with multiple patients near the stadium, structural collapse, stampedes, mass shooting, adverse weather conditions, mass food poisoning, and chemical, biological, radiological, nuclear, and explosives.^10^ In total, 142 medical staff will be providing medical backup in and around ABS. Scattered throughout ABS, 12 medical clinics will commence operations 4 hours before match kick-off and close approximately 2 hours after the final game whistle, once all spectators have vacated the stadium and last mile around it. Each medical clinic will be staffed by a medical doctor and a nurse. Eight HMCAS ambulances will be solely allocated in ABS operations. Six ambulances will be strategically placed in the inner security perimeter near the medical clinics and evacuation lifts. To ensure medical support in the last mile, 2–5 ambulances would be stationed outside the security perimeter for the patients.

HMCAS typically operates two LifeFlight helicopters on a 24-hour basis staffed by Critical Care Paramedic (CCP). On match days, these helicopters will be available to respond to ABS helipad for the rapid evacuation of patients to HGH in the event of a mass casualty incident (MCI). Two of the eight FIFA World Cup Qatar 2022^TM^ stadia have dedicated helipads. These include ABS in the north of the country and Al Janoub Stadium in the south, as they are the furthest from HGH. These helipads will only be used for medical helicopter operations in the event of an MCI were additional CCP capacity is required and to evacuate multiple critically ill/injured patients. The other six FIFA World Cup Qatar 2022^TM^ stadia are located in Doha and in close proximity to HGH (Figure 1).

Three CCP teams are incorporated in the medical entourage at ABS and strategically placed inside and outside the stadium. In the case of an MCI, the dedicated mass casualty staging area will be supplied with two major incident response trucks and a mobile communications truck. Twenty two-paramedic foot patrol teams will be strategically positioned inside and outside of the stadium before, during, and after the match. Other medical resources include paramedics on bicycles and golf carts will patrol the crowded areas.

The Players’ Medical Clinic at ABS is situated at pitch level. The location will facilitate an expedite movement of any player from the field of play (FOP) who may need emergency treatment from the FOP medical team. The two FOP teams comprise of an emergency physician and three paramedics each. Each FOP team will be positioned next to the players’ bench and provide medical backup for half the pitch. All team members on the FOP team have received specialized emergency sports related injury/illness training endorsed by FIFA. If necessary, players will be provided with emergency treatment pitch side before being evacuated by the FOP team to the Players’ Medical Clinic. Once at the Players’ Medical Clinic, the patient will be endorsed to the emergency physician on duty. In consultation with the player’s team physician, the emergency physician will then assess the patient and provide the required emergency treatment. A decision will subsequently be made on the escalation of care potentially required and transportation to the most appropriate receiving healthcare facility.

It is important for health systems and prehospital emergency medical staff to become familiar with such facilities as they become operational because new large capacity stadia have been built over the last few years. To that effect, the HMCAS, which is the national Ambulance Service in Qatar, has conducted several visits and simulation-based exercises in the different FIFA World Cup Qatar 2022^TM^ stadia. The aim of the article is to report on a simulation-based pilot study conducted at one of the largest stadia in the country to compare the air and road transportation times and determine the most effective means of rapid and safe transportation of a patient from ABS to the main trauma center in Qatar. A full-scale simulation-based exercise was planned to realistically test the current emergency procedures and system put in place in relation to the medical evacuation of a critically ill/injured patient.

## Methods

The Scenario for the simulation was based on a player who sustained a fractured lower leg and a concussion during a football match and needed urgent evacuation from the ABS Players’ Medical Clinic near Al Khor to HGH in Doha. The simulated patient was a 23-year-old man. His vital signs were as follows: respiratory rate of 14 per minute, pulse rate of 68 per minute, Glasgow Coma Scale of 15/15, and a blood pressure of 118/71. The same Scenario was performed twice: the first time using a ground HMCAS ambulance and the second time using a LifeFlight helicopter to complete the transportation.

A dedicated HMCAS ambulance stationed in the players’ tunnel near the Players’ Medical Clinic was activated to transport the patient as soon as the emergency physician decided to transfer a player to HGH for further care. For the purposes of this study, the clock was started at the time of activation and stopped when the patient, who was represented by a patient simulator, entered the HGH Trauma Resuscitation Unit (TRU). The two-paramedic team responded with their transport stretcher and standard primary response equipment, including LifePak 15 ECG monitor, medication bag, oxygen bag, portable suction unit, and a medical response bag. At the patient side, the emergency physician endorsed the patient to the paramedic team using the IMIST-AMBO (Identification, Mechanism/Medical complaint, Injuries/Information related to the complaint, Signs, Treatment, Allergies, Medication, Background history, Other information) tool, which is completed in 30 seconds and commonly used at HMCAS.^11^ The patient was then stabilized for transportation, as required, transferred with the transport stretcher, and packaged into the ambulance.

The evacuation of a patient from the pitch to the Players’ Medical Clinic and then to the ambulance is standardized among the eight FIFA World Cup Qatar 2022^TM^ stadia. All medical staff have undergone repeated training to streamline these processes. The procedure for the evacuation of a patient (spectator/workforce) from the stadium is similar for all stadia. Depending on the patient’s location in the stadium, foot patrol teams, as first responders are dispatched to the emergency request. The primary objectives of the medical team are locating the patient, being able to access them effectively, stabilizing the patient by treating any life-threatening emergencies, and then transporting them. Foot patrol teams also escalate emergency care to the CCP. The patient is then prepared and carried to the nearest stationed ambulance for transportation. After secondary triage is completed in the ambulance, a decision is then taken regarding how to evacuate the patient, with potential consideration to use a helicopter in case of an MCI.

The two Scenario occurrences were handled by different teams to prevent recall bias. Prior to starting the clock, all HMCAS teams who participated in the simulation received timely briefings. They were informed that they would be participating in a simulated emergency and were asked to perform as if they were dealing with a real patient in all aspects of care and communication, as per the agreed response protocol to be implemented during the football tournament. HMCAS staff frequently participate in simulation-based exercises.^12,13^ During continuing professional development and as part of their prehospital training, they undergo an extensive three month training program guided by repeated simulation-based exercises, some of which are focused on MCI response. Further, in the build up to the FIFA World Cup Qatar 2022^TM^ HMCAS staff participated in various exercises at the eight stadia, affiliated hotels, and underground metro stations.

**Figure 2.**
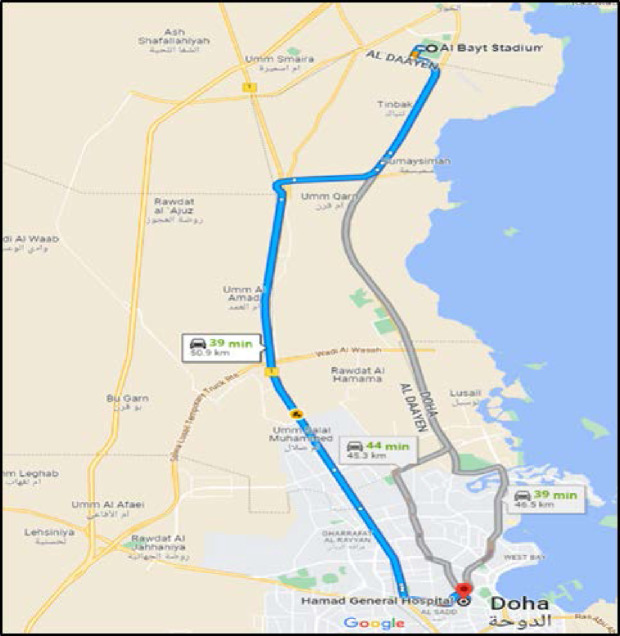
Route used by ground ambulance from Al Bayt Stadium to Hamad General Hospital (picture from maps.google.com).

For the first Scenario (Scenario 1), which involved ground transportation, the ambulance proceeded directly to HGH following nonemergency driving procedures due to it being inappropriate to use emergency driving procedures (with associated risks) in an exercise (respecting speed limits and traffic lights at intersections) (Figure 2). In actual conditions, for Priority (P) 2 emergency driving procedure to hospital with a patient, HMCAS standard operating procedures mandate that the ambulance be driven at the posted maximum road speed limit. Unlike P1 emergency driving procedures, which allow ambulances to exceed the posted speed limit by up to 20 km/hour while still showing consideration for other road users on their way to the scene. P1 emergency driving procedures also allow ambulances to safely pass red traffic signals, provided that it is safe to do so. The designated route via ground from ABS to HGH is on tarmac surfaced multi-lane highways crossing only two major intersections with traffic lights. At HGH, the ambulance parked at the ambulance drop off zone and the patient was moved with the transport stretcher into the TRU, which is when the time was stopped.

**Figure 3.**
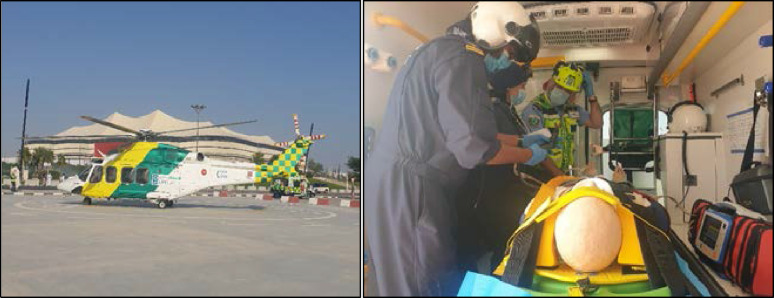
Stadium helipad with LifeFlight helicopter and patient loaded into the ambulance preparing the patient for transport during the simulation-based exercise.

Scenario 2 involved air transportation. A dual process commenced after an evacuation for air transport to HGH was activated. As per ground transport, the dedicated Players’ Medical Clinic HMCAS ambulance was activated. The patient was not transported by ground to HGH, but rather driven following emergency procedures to the ABS helipad. Upon activation, LifeFlight started up in preparation to receive the patient. The ambulance, with the patient onboard, proceeded out of the security perimeter to the helipad near the stadium. According to standard procedure for rotor-wing aircraft operations, the patient was assessed by the flight medical crew, packaged for flight, and hot-loaded into the helicopter.^10^ The aircraft used by HMCAS is an Agusta Westland AW139. The cabin space in the Agusta Westland AW139 helicopter is smaller than the patient compartment space in the HMCAS ambulance but can accommodate two patients. Therefore, patients are stabilized prior to being loaded into LifeFlight for evacuation. Although there are limitations to inflight skill performance, effective patient monitoring and emergency care are still achievable. After that, the helicopter was ready for immediate take-off and routed to the HGH helipad (Figure 3). All primary receiving hospitals in Qatar have dedicated helipads. The helipad at HGH is located on the roof of a five-story car park, which is attached to HGH. Once LifeFlight landed and it was safe to do so, the patient was off-loaded by the paramedical team onto a mobile stretcher. The mobile stretcher was then wheeled to the lifts, taken to the first floor, and transported to the TRU via the adjoining corridor. Just like in the other Scenario, the timer was stopped upon arrival of the patient in the TRU.

For safety reasons and because it would not have been beneficial to any of the exercise objectives, a patient simulator (Laerdal® SimMan ALS) was used for both scenarios rather than someone acting as a simulated patient.^14^ The Scenario for both road and air evacuation was standardized. The patient was a player who had sustained a fractured lower leg and a concussion during a football match. The patient was stabilized by the medical staff in the Players’ Medical Clinic. Ongoing treatment was provided by the practitioners en route to hospital onboard the ambulance and helicopter. In accordance with the objectives of this pilot study, the simulated patient did not need to deteriorate when transported by ambulance and by helicopter and therefore remained stable right through to endorsement by the crew at HGH TRU.

The team facilitating both Scenario occurrences followed the patient the entire time. Time keeping of the exercise was performed by two separate facilitators, noting times at each predefined checkpoint.

**Figure 4.**
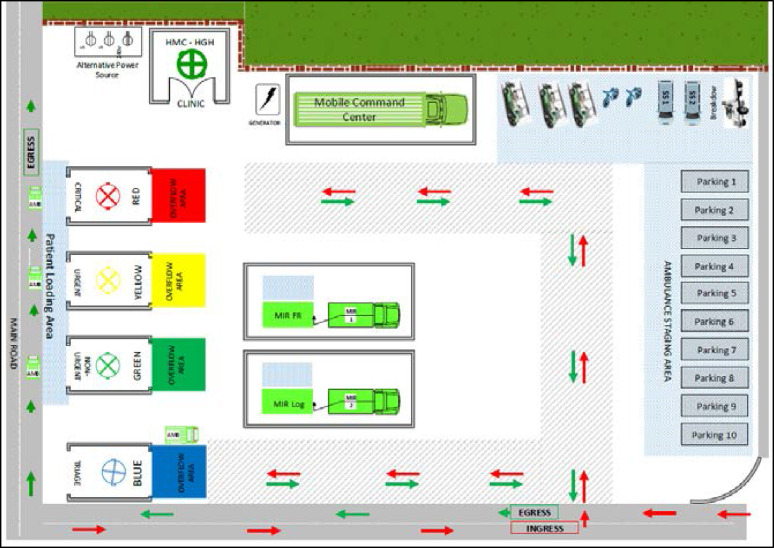
Mass casualty staging area for Al Bayt Stadium.HMC – Hamad Medical Corporation; HGH – Hamad General Hospital; SS – Support Services; AMB – Ambulance.

Although this simulation-based stadium patient evacuation exercise involved one patient, conducting it enabled us to determine the approximate transport time required via both modes of transportation, which is a highly valuable piece of information in case of an MCI, as both would probably be used in such situation. However, each of the eight stadia is equipped to deal with mass casualty related incidents and has a mass casualty staging area (MCA). On match day, this area is dressed with the required MCI resources. Figure 4 shows the MCA for ABS, which follows a similar design as the one, planned for the other seven FIFA World Cup Qatar 2022^TM^ stadia. In the event of an MCI, the emergency medical team at the stadia is guided by the HMCAS Mass Incident Response Handbook. Regular repeated MCI training was provided to all staff at all eight stadia.

On match days, a Venue Operations Center (VOC) will be commissioned into operation 3–4 hours prior to gates opening to welcome spectators. Representatives from each functional area (FA) in the stadium will be represented in the VOC including, but not limited to medical, police, security, spectator services, stadium authority, marketing, Civil Defense, and so forth. Their respective FA representative in the VOC. will closely monitor the execution of the site operational plan for each FA. If any challenge arises, such as a fight breaking out between opposing team spectators, all FAs in the VOC have already rehearsed plans to execute and restore normality with improved communication and teamwork.

## Results

In both scenarios, the dedicated ambulance stationed near the Players’ Medical Clinic was activated promptly via the mobile command center (Figure 3). The paramedics responded immediately to the Players’ Medical Clinic with their primary medical response equipment and transportation stretcher. The paramedic crew response time to the Players’ Medical Clinic for Scenario 1 was 4 seconds quicker (Figure 4). However, patient endorsement, stabilization, and transfer to the ambulance were 2 seconds quicker in Scenario 2. Once the patient was loaded into the ambulance, in Scenario 1, the crew proceeded with nonemergency driving procedures (P2) to HGH following the agreed driving route (Figure 2). The transportation phase for Scenario 1 was 00:28:29 (hours:minutes:seconds) with a total mission time of 00:36:51.

**Figure 5.**
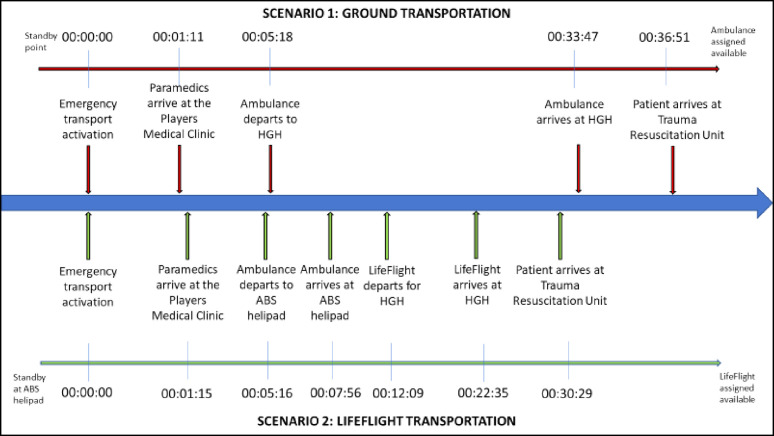
Scenario timeframes for the ground and LifeFlight transportation of the patient to the Trauma Resuscitation Unit in Hamad General Hospital.Note: Time expressed in hours: minutes: seconds. HGH = Hamad General Hospital; ABS = Al Bayt Stadium

For Scenario 2, the ambulance transported the patient directly to the ABS helipad (Figure 5). The transportation time from the players’ tunnel to the helipad through the inner security perimeter fence was 00:02:40. The ambulance safely parked, guided by the flight paramedics. The patient was then endorsed by the ambulance paramedics to the flight crew, who then assessed, packaged, and loaded the patient into the helicopter. Once secured, LifeFlight was airborne toward HGH in Doha. The flight time from the helipad at ABS to the helipad at HGH was 00:10:26.

The transportation phase for Scenario 2 via the LifeFlight helicopter was 63% faster than for Scenario 1 via ground ambulance. However, upon arrival at HGH, the patient arrived marginally sooner at TRU in Scenario 1 as compared with Scenario 2. The overall mission time was thus only 00:06:22 faster in Scenario 2 (LifeFlight helicopter) as compared with Scenario 1 (ground ambulance).

## Discussion

Identifying the fastest and most efficient way to transport a critically ill or injured patient from a mass gathering or mass casualty event is essential to reducing mortality and morbidity. Simulation is increasingly used for systems testing.^21^ In this instance, we identified simulation testing as the most appropriate method to determine the fastest transport time from ABS to HGH TRU, comparing helicopter and ground evacuation. ABS is one of the newly built stadia for the FIFA World Cup Qatar 2022^TM^. Testing of transportation routes from the stadium or surrounding areas was not conducted previously. The simulation was designed around the transportation of a player from the Players’ Medical Clinic. However, other critically ill/injured patients from the stadium precinct may also be transported in a similar fashion to HGH in case of an MCI.

Pretransportation and post-transportation activities were also examined. Pretransportation activity was similar for both scenarios with minimal differences in time despite both scenarios being conducted by two different paramedic teams. However, in Scenario 2, the patient arrived at HGH 00:11:12 faster, but upon landing at HGH it took the patient 00:06:50 longer to arrive in the TRU. This is due to the further distance of the helipad from the TRU as compared with the ambulance drop off zone. Flight paramedics had to descend in a lift from the rooftop to the first floor then pass through an adjoining corridor to reach the TRU.

During the exercise conditions, despite not driving according to usual emergency driving procedures, the ambulance crew did not experience any security or traffic related delays or restrictions. However, during the FIFA World Cup Qatar 2022^TM^, it has been anticipated that traffic volumes would increase in Qatar; Therefore, major road and transportation projects have since taken place and been completed.^16^ The predicted increase in the population by approximately 1.7 million people will demand additional transportation, putting more vehicles on public roads. Road closures around the World Cup stadia will also affect traffic flow and impact on the ambulance transportation time to HGH. These conditions would make the use of P1 emergency driving procedures important during the World Cup period.

In both scenarios, the trauma patient—a patient simulator—arrived at HGH TRU within the Golden Hour from activation of the call. Michaels and colleagues^17^ found that helicopter transports to hospitals improved the survival of trauma patients in their study.^17^ In other studies, helicopter transport has proven advantageous with significantly higher odds of survival in the transport of trauma patients from rural areas as compared with ground ambulance transport.^18^ Shorter transportation times were reported by other studies, which can be of significant benefit to critical patients.^19,20^

Helicopter transportation may however be impeded by a number of challenges including poor weather conditions (normally fine during November and December in Qatar), inadequate landing sites (distance to the stadium), insufficient air traffic control, and poor communication,^21^ all of which have been considered by the FIFA World Cup Qatar 2022^TM^ medical services planning team. Given the findings of the present simulation-based exercise, helicopter transportation of a patient from ABS to HGH TRU for definitive care was only marginally faster than transportation by road ambulance, despite it having to stop at two red lights it encountered on the road.

## Conclusions

Hosting a major sporting event requires careful planning and anticipation of various possible small- and large-scale emergencies. In the present case, we wanted to determine in a realistic manner the time required to transport a patient from the football stadium situated the furthest from the national Level 1 Trauma Center located in Doha comparing two modes of transportation. This simulation-based study has shown that helicopter transfer from the ABS FIFA World Cup Qatar 2022^TM^ stadium to HGH TRU was only marginally faster than by road ambulance. This helped decide on the system to be put in place at the stadium situated the furthest from the national Level 1 Trauma Center and will inform future decision-making to ensure the safe, efficient, and effective transportation of patients who require to be taken to HGH from this location in the shortest time possible.

### Competing interests

The authors have no conflict of interest to declare.

### Funding sources

No funding was received for this research.

### Authors' contributions

The authors confirm contribution to the paper as follows: study conception and design: Authors PG, WT, BM and GA. Data collection: Author PG. Data analysis and interpretation of results: Author PG, WT, BM and GA. Draft manuscript preparation: Author PG and GA. All authors reviewed the results and approved the final version of the manuscript.

(Padarath Gangaram = PG; Wayne Thomsom = WT; Brendon Morris = BM and Guillaume Alinier = GA)

### Acknowledgments

This paper would not have been possible without the support received from Hamad Medical Corporation Ambulance Service management and the staff that participated in the simulation-based exercise testing study. The authors appreciate their contribution to furthering research in the prehospital setting.
